# Low libido despite high normal testosterone levels in a male with an FSH-secreting pituitary macroadenoma

**DOI:** 10.1530/EDM-23-0143

**Published:** 2024-03-22

**Authors:** Randa Ghazal Asswad, Muhammad Ilyas Khan, Catherine Elizabeth Gilkes, Christina Daousi, Sravan Kumar Thondam

**Affiliations:** 1 Department of Diabetes and Endocrinology, University Hospital Aintree, Liverpool University Hospitals NHS Foundation Trust, Liverpool, UK; 2 Department of Neurosurgery, The Walton Centre NHS Foundation Trust, Liverpool, UK

**Keywords:** Adult, Male, White, United Kingdom, Pituitary, Pituitary, Unique/unexpected symptoms or presentations of a disease, March, 2024

## Abstract

**Summary:**

Functioning gonadotroph adenomas with clinical manifestations are extremely rare and the majority of these are FSH-secreting macroadenomas. Clinical symptoms are due to excess gonadotrophins and sex hormones, and these may be present for a long time before the diagnosis of pituitary adenoma is made. We present the case of a 37-year-old Caucasian male with clinical manifestations of an FSH-secreting pituitary macroadenoma. He had sexual dysfunction for a year followed by bilateral testicular pain and enlargement which was initially treated as suspected recurrent epididymitis, but his symptoms did not resolve. He presented a year later with headaches and bilateral superior temporal visual field defects. Brain imaging confirmed a pituitary macroadenoma with optic chiasm compression. Pituitary profile demonstrated an unusually high FSH with high normal LH and normal testosterone level. The patient successfully underwent transsphenoidal hypophysectomy and histology confirmed gonadotroph differentiation and immunoreactivity predominantly with FSH. Gonadotrophin levels and testosterone dropped significantly after surgery, and he was started on testosterone replacement. MR imaging, 2 years post surgery, showed no recurrence of pituitary adenoma. In conclusion, testicular enlargement and hypogonadal symptoms associated with low testosterone levels are recognised features in FSH-secreting pituitary adenomas. Our patient had hypogonadal symptoms but consistently high normal testosterone levels prior to surgery. The reason for low libido despite high testosterone is unclear. Our case highlights the need to suspect such rare underlying pituitary pathology when dealing with unusual combinations of hypogonadal symptoms, testicular enlargement with low or normal testosterone levels.

**Learning points:**

- Functioning pituitary adenomas that secrete excess follicle-stimulating hormone (FSH) are very rare and often present with symptoms related to pituitary mass effect.
- Testicular enlargement alongside sexual dysfunction are commonly reported symptoms amongst male patients.
- Pituitary profile results demonstrate a raised FSH level with either a low, normal, or even high testosterone level which may not always correlate to clinical symptoms.
- Pituitary pathology should be considered in males presenting with unusual combinations of testicular enlargement and hypogonadal symptoms even with normal testosterone levels.

## Background

Pituitary adenomas are benign intracranial tumours arising from the anterior pituitary gland. In the absence of anterior pituitary hormone hypersecretion, these tumours are defined clinically as non-functioning pituitary adenomas (NFPA). Histological classification based on the cell lineage transcriptions factors, indicate that more than 70% of these NFPAs express gonadotrophins or their transcription factors on immunohistochemistry. However, the vast majority of these do not secrete excess gonadotrophins at clinically relevant levels and are therefore classified as silent gonadotroph adenomas (1). Functioning gonadotroph pituitary adenomas that secrete excess follicle-stimulating hormone (FSH) and luteinizing hormone (LH) leading to clinical manifestations are extremely rare with the literature detailing only few reports. Of these, FSH secreting adenomas are the predominant type and present as macroadenomas (>1 cm in size). The clinical features in pre-menopausal females include menstrual irregularity and ovarian hyperstimulation syndrome. In males, symptoms of sexual dysfunction, and testicular enlargement are well recognised (2, 3). The diagnosis usually comes to light when patients present with symptoms related to hypopituitarism or due to compression of surrounding anatomical structures by the tumour leading to visual disturbances. Unusually high gonadotrophins provide the clue to diagnosis in males, but diagnosis can be difficult in females (particularly post-menopause) due to variable FSH and LH levels.

This report describes the case of a young man with an FSH-secreting pituitary macroadenoma who presented with a distinctive combination of symptoms and biochemical parameters resulting in diagnosis only occurring late in the course of the disease. It thus aims to highlight the important clinical factors for consideration in diagnosis of this rare condition.

## Case presentation

We present the case of a 37-year-old, Caucasian male with a history of sexual dysfunction and loss of libido for 1 year. Other than being obese, he had no other significant past medical history. The first presentation of his condition was to a primary care clinic with a 6-month history of bilateral testicular pain, swelling of both testes and sexual dysfunction. Blood tests revealed normal testosterone levels; thus, he was referred to a urologist for further assessment of his symptoms. Clinical examination and investigation in the urology clinic at that time revealed bilaterally enlarged testes (>30 mL in volume), particularly worse on the right side, with no pathological features identified on ultrasound imaging of the testes. The patient received medical management for suspected recurrent epididymitis but there was no resolution of his symptoms. A year later, he presented to the emergency department with a 1-month history of generalised headaches, dizziness and visual disturbances. Brain imaging at this stage led to the diagnosis of a large pituitary macroadenoma compressing the optic chiasm. Ophthalmological examination demonstrated bilateral temporal field defects with left sided superior temporal quadrantanopia and right sided incomplete temporal hemianopia. Visual acuity was normal.

## Investigation

**Figure 1:**
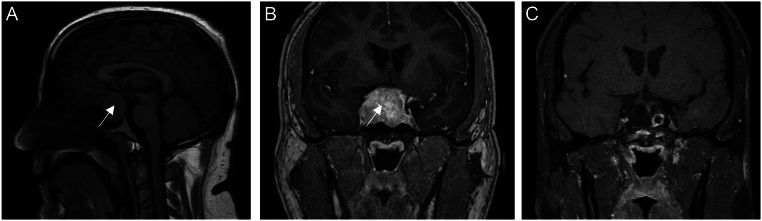
(A and B) MR imaging pre-operatively in the sagittal and coronal planes demonstrated a 3.0 × 2.5 × 3.0 cm sellar and suprasellar region mass. There is optic chiasm compression. No other intracranial pathology was noted. (C) Post-operative pituitary imaging demonstrates no evidence of residual disease or recurrence.

Magnetic resonance imaging (MRI) of the brain confirmed a large pituitary macroadenoma measuring 3.0 × 2.5 × 3.0 cm with optic chiasm compression (Fig. 1A and B). Ultrasound of the testes performed twice showed normal radiological appearances of the testes. DEXA scan revealed high normal bone mineral density at the spine (1.670 g/cm^2^, *T* score: 3.7, *Z* score: 3) and femur (1.207 g/cm^2^, *T* score: 0.9, *Z* score: 0.6).

A pituitary profile demonstrated an unusually high FSH of greater than 200 IU/L (normal reference range 0.7–11.1), high normal LH of 6.0 IU/L (0.8–7.6) and normal testosterone levels of 21.8 nmol/L (8.5–29.0). Prolactin, cortisol and TSH were in normal limits (Table 1). Furthermore, he had mild polycythaemia with a haemoglobin of 181 g/L (130–175) and a haematocrit of 0.51 L/L (0.4–0.52). Review of previous blood results indicated his haemoglobin was above the upper limit of normal for the previous 10 years.

**Table 1:** Hormone test results pre and post treatment.

Test	RR	Pre-operative	Post-operative		
		At diagnosis	Day 1	2 months	6 months
FSH, IU/L	0.7–11.1	>200	18.0	3.6	1.9
LH, IU/L	0.8–7.6	6.0	<0.1	<1.5	<0.1
Testosterone, nmol/L	8.5–29.0	21.8	–	0.90	21.4*
Prolactin, mIU/L	0–350	307	–	106	259
Cortisol, nmol/L	140–700	152	–	489	184
TSH, mIU/L	0.30–5.00	1.22	–	1.16	1.04
Free T4, pmol/L	8–22	25.1	–	–	13.8
Free T3, pmol/L	2.5–6.5	7.1	–	–	3.9

*Patient on testosterone hormone replacement.

RR, reference range.

## Treatment

The patient was referred to a joint pituitary clinic and successfully underwent transsphenoidal hypophysectomy with near complete resection of the pituitary adenoma. Subsequent annual follow-up MRI for the next 2 years have not shown evidence of recurrence of the pituitary tumour.

## Outcome and follow-up

The patient’s vision improved immediately after pituitary surgery. On day one, post-pituitary surgery, his FSH levels dropped to 18 IU/L and LH was undetectable (Table 1). Histology demonstrated gonadotroph differentiation and immunoreactivity predominantly with FSH but also LH and prolactin stains. Gonadotrophin levels and testosterone dropped significantly within 8 weeks after surgery indicating hypogonadotrophic hypogonadism. His symptoms of hypogonadism had also worsened, and he was started on testosterone replacement (1000 mg testosterone undecanoate injections, once every 12 weeks) which he continues till present. He continues to have symptoms of testicular pain but at a reduced intensity. The latest MR imaging of his pituitary gland at 2 years post surgery shows no significant residual or recurrence of the pituitary tumour (Fig. 1C). His polycythaemia has also resolved with haemoglobin now in the normal range.

## Discussion

The hypothalamic–pituitary–gonadal axis in males drives testosterone secretion and spermatogenesis. Negative feedback from testosterone inhibits LH secretion directly at the pituitary level and FSH secretion indirectly through suppression of gonadotropin releasing hormone at the hypothalamus level. This feedback mechanism is lost in functioning gonadotroph adenomas. The clinical challenge of diagnosis lies in the fact that there is a wide variation in gonadotropin hormones and testosterone levels with these tumours. When testosterone levels are low, the biochemical picture may mimic primary hypogonadism. Brain imaging may not always be performed unless there is clinical suspicion based on symptoms and an unusual pattern with regards to the pituitary hormone profile.

In males, testicular enlargement, hypogonadism and mass effects of the tumour are the main manifestations of functioning gonadotroph pituitary adenomas (2). Pre-operative hormonal profiles vary with many cases showing a raised FSH level with either a low, normal, or even high testosterone level (Table 2) (3, 4, 5, 6, 7, 8, 9, 10). In most cases described in the literature, LH levels were normal; however, in the few cases where LH levels were raised, this led to higher testosterone levels.

**Table 2:** Summary of reported cases of gonadotroph secreting pituitary adenomas in adult male subjects.

Study/case	Age, years	SD at presentation	Hormonal results at diagnosis					
			FSH, U/L		LH, U/L		Testosterone	
			Result	RR	Result	RR	Result	RR
Our case	37	Present	↑		N		N	
Heseltine *et al.* (3)								
a)	41	Absent	↑40	<7	N 4.2	<7	N 19 nmol/L	11–30
b)	41	Absent	↑>40	<7	N 8.8	<7	↑33 nmol/L	11–30
c)	69	Present	↑27	<7	N 8.5	<7	↓6 nmol/L	11–30
d)	55	Present	↑>20	<7	N 4.3	<7	↓	
Pigny *et al.* (4)	47	Absent	↑29.2	1–7	N 1.0	0.5–10	N 3.35 ng/mL	3–10
Dizon & Vesely (5)	61	Absent	↑72.48	0–20	↑31.65	0–25	↑15.24 ng/mL	3–10
Usui *et al.* (6)	40	Absent	↑27.7	0–20	N		N	
Dahlqvist *et al.* (7)	56	Present	↑27	0.7–11	N 1.0	0.8–7.6	↓5.0 nmol/L	9.4–37
Chamoun *et al.* (8)	45	Absent	↑35.6	1.5–12.4	↑10.8	1.7–8.6	↑>1500 ng/dL	300–890
Thakker *et al.* (9)	48	Absent	↑32.7	1–10	↑11.5	1–7	↑1647 ng/dL	220–1000
Cote *et al.* (10)								
a)	45	Absent	↑27.8	0.9–15.0	N 4.5	2.4–5.9	N 253.9 ng/dL	165–1194
b)	52	Absent	↑159.1	1.3–19	N 4.2	1.7–8.6	N 880 ng/dL	250–1100
c)	71	Present	↑125.3	1.5–12.4	N 2.5	1.7–8.6	↓48.0 ng/dL	193–740
d)	62	Present	↑25.0	1.5–12.4	N 2.2	1.7–8.6	N 233 ng/dL	193–740
e)	64	Absent	↑33.2	1.5–12.4	N 4.5	1.7–8.6	N 364 ng/dL	348–1197

↑, increased; ↓, decreased; N, normal; RR, reference range; SD, sexual dysfunction.

Heseltine *et al.* (1989) reported four male patients with FSH-secreting pituitary macroadenomas with testicular enlargement. All patients had raised FSH levels pre-operatively with variable testosterone levels. Interestingly, those with normal testosterone levels reported no loss of libido, whilst patients with low testosterone levels reported additional sexual dysfunction symptoms (3). None of the patients reporting low libido at presentation had a high normal testosterone level except for one patient reported by Cote *et al.* (2016), which is similar to our case (10). Our patient had testicular enlargement and consistently high normal testosterone levels prior to surgery and polycythaemia for many years before presentation. This may be due to concomitant hypersecretion of both FSH and LH from his pituitary adenoma. The reason for low libido despite a high normal testosterone level is not entirely clear. However, the other physiological effects of testosterone were preserved such as erythropoiesis leading to mild polycythaemia and a high normal bone densitometry.

It is important to differentiate between functioning gonadotroph adenomas and clinically NFPAs. Functioning adenomas would require pituitary surgery earlier to minimise the adverse effects of hormone hypersecretion even in the absence of tumour compressive effects on surrounding structures. Surgery would also be intended for near total resection of functioning tumours. In NFPAs, the aim of surgery is to relieve the compressive effects and a complete removal of the pituitary tumour may not be undertaken if the tumour is extensive or present in technically inaccessible areas.

In males, bilateral testicular hypertrophy in the absence of testicular pathology and sexual dysfunction independent of testosterone levels should prompt endocrinological work-up for suspicion of these rare gonadotrophin secreting pituitary adenomas. Testicular hypertrophy is driven by FSH induced hypertrophy of the seminiferous tubules (3). Early diagnosis and management of these tumours would alleviate symptoms while avoiding other unnecessary treatments for testicular pathology. Our patient had initially presented to urology and was treated for suspected epididymitis repeatedly, yet there was no significant testicular pathology identified on repeated ultrasound scans of his testes. In primary care clinics, gonadotrophin levels may not always be included as first line tests alongside testosterone in the investigation of hypogonadal symptoms. A normal testosterone level in most cases would not usually lead to further endocrine investigations. Thus, clinicians should be encouraged to perform gonadotropin levels alongside testosterone when interpreting these constellations of signs and symptoms.

Our patient differs from most other cases reported in the literature as he had hypogonadal symptoms with consistently high normal testosterone levels prior to surgery. The timing of diagnosis is in keeping with other cases described where diagnosis is often made soon after presentation with mass effect symptoms of the tumour, rather than earlier clinical manifestations. This case highlights the need to suspect such rare underlying pituitary pathology when dealing with unusual combinations of hypogonadal symptoms, testicular enlargement and normal testosterone levels. In such cases, referral to a pituitary endocrine team is essential to provide optimal management of the pituitary tumour.

## Declaration of interest

The authors declare that there is no conflict of interest that could be perceived as prejudicing the impartiality of the study reported.

## Funding

This work did not receive any specific grant from any funding agency in the public, commercial or not-for-profit sector.

## Patient consent

Written informed consent for publication of this case report was obtained from the patient.

## Author contribution statement

RGA collected data and composed the manuscript, alongside senior co-author MIK. CEG, CD and ST were involved in this patient’s clinical care and provided critical revision of the manuscript.
